# Affirmative Action in Criminal Justice

**DOI:** 10.1093/ojls/gqaf017

**Published:** 2025-06-17

**Authors:** Benjamin Ewing

**Keywords:** criminogenic disadvantage, fair moral opportunity, moral fortification, affirmative action, sentence mitigation, *Gladue*

## Abstract

Even if a hiring process is merit-based and non-discriminatory, it may still fail to ensure substantive fairness if some applicants lacked a fair opportunity to develop their qualifications to compete. A familiar potential remedy for the problem is ‘affirmative action’, in the sense of preferential treatment for job candidates who lacked a fair opportunity to develop their job qualifications. I defend two analogous contentions about criminal justice. Even if criminal sentencing is formally fair—ie free of discrimination and bias—it may still be substantively unfair because some disadvantaged offenders have lacked a fair opportunity to develop their capacities and structure their choice environments to fortify themselves against resorting to crime. And the criminal justice system might implement a form of ‘affirmative action in criminal justice’ by mitigating the punishment of offenders who are culpable for crimes but lacked a fair opportunity to avoid becoming so.

## 1. Introduction

In Canada’s most recent census in 2021, only about 5% of its population identified as Indigenous. Yet, that same year, the percentage of federal prisoners in Canada who were Indigenous reached 32%.^1^ Likewise glaring is the overrepresentation in US custody of Black Americans, who are estimated to make up 14% of US residents but about 41% of those in jail and prison.^2^ Racial disparities in punishment are especially salient. But, in any given case, they are likely to be explained in substantial measure—even if far from entirely—by a correlation between race and class.^3^

The disparate impact of punishment on disadvantaged groups is easy to critique to the extent that it is the outcome of a discriminatory criminal justice system that punishes crimes differently based on the race, class or marginalised social status of who commits them—whether because of biases among decision makers or because the socioeconomically disadvantaged have fewer resources with which to defend themselves against criminal prosecution. Yet the overrepresentation among prisoners, probationers and parolees of a disadvantaged group—eg Black Americans in the United States, Indigenous people in Canada and, more generally, people who are poor, under-educated, under-employed, socially marginalised or intergenerationally traumatised by historical injustice—might, in any given case, be explained at least as much by higher levels of criminal offending by members of that group as by unequal treatment of them by the criminal justice system.^4^ We should expect elevated rates of offending to play a significant role in explaining unequal criminal justice outcomes—not because invidious bias and discrimination in criminal justice systems are things of the past, but because theory and experience suggest that concentrated disadvantage amid affluence is criminogenic.^5^

That raises the question: is greater punishment borne by a disadvantaged group still potentially unjust, even to the extent that it can be traced to greater criminal offending by members of the group (which makes them unequally situated), or only to the extent that their greater punishment can be traced to bias or discrimination (which causes them to be treated unequally even when equally situated)? I argue that we can answer that question more perspicuously if we construct an analogy between it and a structurally similar question whose contours and potential answers are better understood: can adverse, unequal employment outcomes faced by a disadvantaged group be unfair, even to the extent that they can be explained by lesser education and training for valuable jobs making members of the group less qualified on average, or only to the extent that the outcomes can be explained by bias and discrimination leading to the unequal treatment of the equally qualified?

One familiar answer to that latter question is that to be unjust, disparities in employment outcomes need not be traceable to unequal treatment of equally qualified applicants (ie unfairness in a formal sense). They may also be traceable to the fact that disadvantaged groups have lacked fair opportunities to get the education and training necessary for them to have a fair chance to compete in the labour market (ie unfairness in a substantive sense).^6^ Moreover, when that is the case, a potential remedy is a particular form of ‘affirmative action’ whereby employers endeavour to correct for certain job applicants’ lack of fair opportunity to develop their qualifications, by treating it as a consideration in favour of their selection.^7^

In this article, I reflect on the possibility of an analogous diagnosis of, and response to, the greater punishment that disadvantaged groups may bear owing to greater criminal involvement (rather than or in addition to unequal treatment by their criminal justice systems). I do so by constructing an analogy between formal and substantive fairness in employment and punishment, and explaining how it points to the possibility of a form of ‘affirmative action in criminal justice’: mitigating the punishment of those who are culpable for crimes (and in that way ‘qualified’ for punishment) but who have lacked a substantively fair opportunity to avoid punishment, because they have not had fair background opportunities in life to cultivate their capacities and dispositions, and structure their choice environments, in ways that facilitate reliably avoiding crime.^8^ Along the way, I reflect on the puzzle of why such an analogy and proposal have rarely been countenanced, let alone subjected to sustained philosophical scrutiny.^9^

In section 2 of the article, I clarify the type of ‘affirmative action’ of interest to me and my reasons for focusing on it. In section 3, I discuss ‘qualification’ for employment and punishment. I show that much as a lack of fair background opportunity in life may help to explain why a person has failed to accrue the positive qualification of being fit for rewarding employment, so too may it help to explain why a person has ‘succeeded’ in accruing the negative ‘qualification’ of being fit for criminal punishment. In section 4, I turn to ‘fair opportunity’ in employment and punishment. I draw upon the more familiar idea of fair opportunity to cultivate one’s capacities, talents and skills, to help explicate the novel but analogous idea of fair opportunity in life to buttress the reliability with which one adheres to the law and avoids culpable crime. And in section 5, to illustrate what affirmative action in criminal justice could look like in practice, I present as a case study the *Gladue* principles, which govern the sentencing of Indigenous people in Canada.

Before proceeding further, an introductory caution is in order. Many potential objections to affirmative action in criminal justice, as I conceive of it, can be derived from familiar objections to affirmative action in hiring. Though I mention a few of these in concluding remarks, I do not work them out in any detail, let alone seek to rebut them. As I see it, if readers object to affirmative action in criminal justice for reasons that mirror their objections to affirmative action in hiring, that reinforces rather than undercuts the article’s core argument. For my aim is not to offer a substantive moral defence of affirmative action in criminal justice so much as simply to make the case that the idea can help us to better answer the motivating question with which the article began: might it be wrong that those we punish come disproportionately from socially disadvantaged groups, even to the extent that it reflects greater criminal offending by their members? Even those who would answer ‘no’ to that question should benefit from this article’s effort to make sense of why others might answer ‘yes’—and what the logic of the yea-sayer’s position would suggest a criminal justice system ought to do about it.^10^

## 2. Qualification and Fair Opportunity in Employment and Punishment

As HLA Hart famously argued, whatever may be the ‘general justifying aims’ of punishment, distinct ‘principles of distribution’ may limit whom we should punish and how much.^11^ We typically assume that our pursuit of such goals as reducing and condemning crime should be subject to various moral side constraints, such as that punishments should not be disproportionate to the gravity of crimes for which they are imposed, and that comparably serious and culpable crimes should be punished the same or similarly. Even those retributivists who take the justifying aim of punishment to be giving people their retributive deserts very often recognise a crucial moral side constraint on the pursuit of that goal—namely, that punishment generally ought only to be imposed for conduct proscribed in advance,^12^ so as to uphold the rule of law and give individuals a chance to economise on virtue and avoid crime because of its prohibited status (and not merely its moral wrongfulness).

There is a version of the same point to be made with respect to employment. Moral considerations constrain, and potentially compete with, employers’ pursuit of their aims in hiring employees. Most obviously, employers are morally and legally prohibited from engaging in invidious discrimination based on certain protected characteristics of applicants (eg their race, sex and religion). And as TM Scanlon has suggested, it is plausible that for an institution creating unequal outcomes to be justified there must be not just procedural fairness in the process by which the institution facilitates unequal outcomes but also fair ‘substantive opportunity’—ie ‘no wrong involved in the fact that [some people] did not have the necessary qualifications or other means to do better in this process’.^13^ Employers may strive not only to respect *formal* fairness in their application processes, but also to realise *substantive* fairness, through a particular form of affirmative action. They may do so by treating it as a consideration in favour of an applicant that she lacked a fair opportunity to develop her capacities, skills and talents, and thereby compete for the job.^14^

This form of affirmative action in employment—which broadly corresponds to what Kasper Lippert-Rasmussen has labelled affirmative action grounded in the ‘equality of opportunity’ rationale^15^—is what I have in mind as I develop the case for an analogous form of affirmative action in criminal justice. Whereas I take ‘affirmative action’ in a general sense to refer to practices that accord preferential treatment, in an allocation of benefits or burdens, to members of some group (especially a group whose members have historically been less likely than members of other groups to accrue those benefits or avoid those burdens), the specific type of affirmative action in hiring that is of interest to me in this article has two key features that distinguish it from other forms of affirmative action. First, it involves a partial sacrifice of goals the employer exists to serve, as reflected in the job qualifications sought. Second, the moral consideration against which the goals justifying an employer’s existence are to be traded off is substantive fairness in the hiring process. Though there are multiple possible ways to transplant this kind of affirmative action into the criminal justice system, I focus on mitigation at sentencing for offenders who lacked a fair opportunity to avoid the behaviour that made them ‘qualified’ for punishment given punishment’s justifying aims.^16^

In employment, race-based affirmative action is often defended as a way of achieving a number of goals distinct from substantive fairness—including correcting for formal discrimination and bias in standardised measures of ‘qualification’ (eg biases against applicants whose social backgrounds put them at a disadvantage in their ability to craft effective applications or perform well in interviews).^17^ Forms of affirmative action in criminal justice could theoretically be developed and defended on analogous grounds. To the extent that certain groups face bias or discrimination in the application of the law, putting a thumb on the scale against the policing, prosecution or punishment of their members might be conceived simply as a way of approximating the outcomes that would arise from unbiased, formally equal treatment. And were we to bring criminal prohibitions and the criminal law’s conception of culpability into line with critical morality, we might see that punishment of certain disadvantaged offenders does not serve the justifying aims of punishment as effectively as we might have thought, because their crimes are partially excused, which makes them less important to condemn and discourage.^18^

As various theorists have recognised, it is intuitive that offenders sometimes face circumstances or conditions that are not extreme enough that they should defeat liability but which should be taken partially to excuse or mitigate culpability, because they exist somewhere on continuums of severity that lead, in their limit cases, to full defences.^19^ To avoid differentiating criminal offences in too fine a manner, to guide conduct clearly and to maintain a rigorous standard of responsibility, it makes sense to have relatively coarse-grained offence categories and high thresholds for full defences to liability. At the same time, doing so inevitably leaves more fine-grained distinctions in people’s degrees of culpability (and in the fairness of their opportunity to avoid their crimes), which are precisely the kinds of concerns that are central to the sentencing stage, rather than the guilt-determination phase, of the criminal process. So there should be little trouble in recognising that even if an offender is ‘qualified’ for punishment in the sense of being liable to be punished, it might be unfair to subject him to punishment as harsh as his crime would presumptively warrant. For he might, owing to partially excusing conditions, have lacked a fully fair opportunity to avoid becoming guilty of his crime.

That line of thought leads us to a version (but one more minimalist than what I have in mind) of fairness-based affirmative action in criminal justice: mitigating the punishment of disadvantaged offenders as a way of making punishment proportionate to a nuanced assessment of their reduced culpability (and concomitantly reduced ‘qualification’ for punishment). Construed in that way, however, affirmative action in criminal justice would be too narrow and limited, in two important respects. First, it would involve nothing more than a familiar, widely shared ideal of proportionate sentencing, which can be understood and justified easily, without recourse to any novel notion such as ‘affirmative action in criminal justice’. Second, for obvious reasons I address momentarily, the familiar ideal of calibrating a sentence in proportion to an offender’s degree of culpability offers us meagre resources for answering the vexing question that our inquiry into the possibility of affirmative action in criminal justice was meant to help us answer: when disadvantaged groups bear an outsize share of punishment, is this potentially unfair or unjust even to the extent that it reflects greater criminal involvement by members of those groups?

A comparison with employment can help us to see just how much we overlook and forgo if we restrict affirmative action in criminal justice to punishing in proportion to a nuanced assessment of an offender’s culpability. In assessing job candidates’ qualifications, employers have good reason to start by considering standardised markers of achievement and potential, such as: past employment experience; licences, degrees and training programmes completed; performance on tests and in courses; and so on. These are standardised measures of ‘qualification’ for a job that can be usefully compared with the criminal law’s standardised measures of ‘qualification’ for punishment—namely, its general prohibitions of certain forms of behaviour. Much as states have good reasons to supplement course-grained, binary liability rules with fine-grained, scalar assessments of culpability at sentencing, employers have good reasons to go beyond crude measures of job qualification that might be listed in an advertisement or résumé—especially metrics that may be to some degree biased against certain groups.

Yet, it would be absurd—and harmful to those in need of help—to assume that because standard measures of job qualification may contain biases against unfairly disadvantaged groups, once qualification is properly assessed we shall find their members are not any less qualified for desirable employment than members of other groups. To take that position would be to assume away the sad reality that among the most powerful mechanisms of unfair disadvantage in employment are background social conditions that deprive many people of fair opportunities to become qualified in a genuine and not merely superficial sense. A large part of why poverty, lack of educational opportunity, and intergenerational familial and community trauma are social disadvantages is precisely that they deprive their victims of fair opportunities to develop the capacities, skills and talents they would need to be strong candidates for valuable employment.

Why, then, have theorists of fair opportunity to avoid crime focused on ways in which an unfairly disadvantaged background may diminish an offender’s true ‘culpability’ (much as it may enhance a job applicant’s true ‘merit’), neglecting the possibility that a background of unfair disadvantage may also deprive a person of a fair opportunity to avoid incurring genuine culpability (much as such a background may deprive a person of a fair opportunity to develop her job qualifications, and not just an opportunity to have her true qualifications assessed without bias)?

Several answers suggest themselves. First, there is a reasonable desire not to highlight or dwell on disparate rates of culpable offending across groups—particularly racialised ones—because of the potential for this both to reinforce and to divert attention from institutional bias and discrimination in assessments of criminal guilt and culpability. Second, precisely because criminal blame carries such a moral stigma, it is difficult to imagine how a person could be worthy of it if she lacked a fair opportunity to avoid it. Third, it might appear that the capacities, skills and talents needed for rewarding employment must be cultivated through education, training and socialisation over long periods of time, whereas all that is needed to have a fair opportunity to avoid crime is simply the absence of immediate coercive pressures and emergency circumstances that might furnish criminal defences or partial excuse variants thereof.

The first worry—stereotyping—is real and significant, but appears to apply with comparable force in the context of hiring. Although the stakes may be lower in hiring than in punishment, we understandably wish to avoid stereotyping individuals from unfairly disadvantaged backgrounds as less qualified for desirable employment, much as we seek to avoid stereotyping them as engaging in culpable crime more frequently. The second and third issues, however, are the distinct stigma that attaches to being culpable for a crime (which arguably does not attach to the failure to qualify for valuable employment) and the kind of social support needed to cultivate job qualifications (which is arguably not required, to avoid wrongdoing that is justifiably criminalised). Those are apparent *asymmetries* between qualification for punishment and qualification for employment, and between the fair opportunity necessary or conducive for cultivating job qualifications and the fair opportunity necessary or conducive for refraining from crime.

Nevertheless, I contend that in much the way that the substantive fairness of the allocation of jobs depends on the fairness of people’s background opportunities to cultivate job qualifications, so too the substantive fairness of the allocation of punishment depends on the fairness of people’s background opportunities to fortify themselves against pressures that might lead them to commit culpable crimes. To vindicate that claim, I need to show two things: (i) in spite of the special stigma of criminal culpability, it remains possible for a person to incur this ‘qualification’ for punishment despite having (in some importance sense) lacked a fair opportunity to avoid doing so; and (ii) fair opportunity to avoid qualifying for punishment can be meaningfully compared with fair opportunity to qualify for employment, notwithstanding the differences between them. Making the case for those two propositions is therefore the task of the next two sections of the article.

## 3. Qualification for Employment and Punishment

In choosing among those candidates who exceed the threshold of qualification, an employer may treat a candidate’s lack of fair opportunity to develop her qualifications as a ‘plus factor’ that, all else being equal, favours hiring her. In determining how much to punish, the state can, analogously, mitigate the punishment of offenders who lacked a fair opportunity to avoid the culpability for crime that has qualified them for punishment. It is possible to take account of people’s opportunities to cultivate positive qualifications (ie for employment) and to avoid incurring negative ones (ie for punishment) without hiring the unqualified, and without punishing the innocent or letting the guilty off entirely.

One important asymmetry, however, may help to explain why, despite the salience of affirmative action aimed at substantive fairness in hiring, the possibility of an analogous form of affirmative action aimed at substantive fairness in punishment has been overlooked: a special moral stigma attaches to qualifying for punishment that does not attach to failing to qualify for employment. Being unqualified, or less qualified, for a job carries no necessary implication that one is morally at fault for being so. It is therefore straightforward that a person could be unqualified, or less qualified, for a job at least partly as a result of lacking a fair opportunity to develop her qualifications. By contrast, precisely because the criminal culpability needed to qualify for punishment carries the moral stigma of blameworthiness, it is puzzling how a person could rightly accrue it, except to the extent that she had a fair opportunity to avoid doing so.

How, then, might one be ‘qualified’ for punishment despite having lacked a fair opportunity to avoid becoming so? Recall that a person is qualified for punishment, in the sense in which I am using the term, when and to the extent that her conduct is a good candidate for punishment from the standpoint of its justifying aims. Hence, to show that it is possible to become qualified for punishment despite lacking a fair opportunity to avoid it, we must do two things. First, we must derive a working conception of qualification for punishment from reflection on the justifying aims of punishment. Second, we must explain how it is possible for a person to incur qualification for punishment in the relevant sense, despite unfair obstacles to avoiding such qualification.

Despite salient, reasonable disagreements over the justifying aims of punishment, it appears overdetermined that if punishment is directly and reliably to pursue its justifying aims, conduct must be punishable only when and to the extent that it ought to be both discouraged and condemned. Insofar as the justifying aim of punishment is to give people their retributive deserts, punishment can hardly be appropriate absent blameworthy conduct. And insofar as the justifying aim of punishment is to condemn and discourage conduct that ought to be condemned and discouraged, punishment will only directly and reliably serve that aim to the extent that it is applied to individuals who have engaged in conduct that truly ought to be discouraged and condemned.^20^

Moreover, although it is also controversial how best to conceptualise criminal culpability, it likewise appears overdetermined that criminal culpability presupposes—at the very least—conduct fit not only to be discouraged but also condemned. Indeed, two of the leading contemporary theories of criminal culpability can be seen precisely as competing accounts of what makes conduct fit not only to be discouraged but also condemned. One such theory treats criminal culpability as a function of the degree to which an offender manifested attitudes of insufficient regard for other people and their legally protected rights and interests—which depends on the risks the offender’s conduct ran and the reasons for running them.^21^ Another theory treats criminal culpability as a function of the gravity of an offender’s failure to act in accordance with the underlying reasons—from the law’s point of view—not to commit the crime.^22^ So, although I cannot hope to resolve here the many debates surrounding how best to conceptualise criminal culpability, it should not be overly controversial to operate on the assumption that what ‘qualifies’ a person for punishment is criminal culpability, understood in terms of how gravely an offender’s conduct departed from adequate concern for other people.^23^

What determines the degree to which an offender manifests insufficient regard for other people’s legally protected rights and interests, and the degree to which she fails properly to respond to the legal reasons against her crime? Each depends on the strength of the relevant reasons against the conduct constituting her offence and the strength of the relevant reasons in favour of that conduct. For instance, as Alexander, Ferzan and others have argued, reckless wrongdoing can be understood as consciously running a risk when that risk is unjustified because it outweighs in significance the reasons for running it.^24^ More generally, criminalisation of an act reflects a judgment that the reasons against it at least presumptively outweigh whatever reasons an agent might have to perform that act.

Some special obstacles to avoiding wrongdoing alter the degree to which an agent manifests insufficient concern for others or departs from the appropriate weighing of reasons that the criminal law implicitly demands of its subjects. For example, driving a motor vehicle much faster than the legal speed limit and failing to obey ordinary traffic rules may be reckless in ordinary cases. Yet, if a passenger is suffering from a life-threatening medical emergency, what would ordinarily be reckless driving may be supported by the balance of reasons in the circumstances; the risk that the passenger might die may simply match or outweigh the risks the driver is running by flouting traffic rules. Other legal justifications or excuses, such as self-defence and duress, can likewise generally be understood as altering the balance of reasons for and against the conduct constituting the would-be crime. Self-protection, for instance, creates a good reason to harm a culpable aggressor, and the aggressor’s culpability for creating the threat and opportunity to avoid doing so weaken his claim against being harmed defensively. And when an agent assists in the commission of a crime under duress—ie because she is coerced into doing so by a credible threat of immediate death or serious injury, to which even a person of reasonable firmness might succumb—the importance of protecting herself from imminent harm and the lack of alternative means of doing so combine to create a good reason to accede to the threat (even if, as is often thought, this reason only excuses and does not justify the *prima facie* criminal conduct).^25^ Finally, if an adolescent or mentally ill person commits an assault but, owing to her youth or mental condition, underestimates the magnitude of the unjustifiable risk she is running to her victim’s legitimate interests, this makes her crime manifest less deficient regard for other people, which diminishes her culpability.^26^

Yet there can also be obstacles to avoiding wrongdoing that do not reveal it to manifest any less deficient a degree of concern for other people than such wrongdoing would ordinarily manifest. (And, as I argue in the next section, such obstacles can be unfair.) Human beings are not all equally well positioned to give others their due by acting in accordance with the practical reasoning demanded by the criminal law. This is true not only because of immediate pressures to offend and differences in people’s capacities to recognise and respond to the reasons against crime, but also, perhaps even more importantly, because of differences in their opportunities to develop their capacities and to structure their social circumstances to fortify themselves against psychic and environmental pressures that lead people to manifest inadequate regard for other people.

If an offender faced special obstacles to avoiding his crime, even if those obstacles did nothing to reduce the deficiency of regard for others that his crime manifested, such obstacles may still be an appropriate ground for mitigating his punishment. Indeed, there are familiar factors that are commonly, and plausibly, thought to be sound bases for mitigating an offender’s punishment, even if they fail to reduce the degree of insufficient concern for other people manifested in the offender’s crime. Consider, for instance, various forms of diminished capacity and psychological compulsion. As we have seen, diminished capacity is sometimes simply an explanation of why a person lacked (or had a lesser) *mens rea* (as when, for example, an adolescent is unable fully to appreciate the nature or magnitude of the risks of his conduct). But diminished capacity can also make it more difficult for a person to recognise and respond to the good reasons not to commit an offence. For example, even an adolescent who fully appreciates the factual risks of her conduct may fail fully to appreciate the normative importance of those risks, or she may have a harder time aligning her conduct with what the law expects of her. And this may, in turn, be a consequence of the fact that as a young person she faces stronger impulses toward immediate pleasure seeking and has less well-developed capacity for self-control. The psychological compulsions faced by those in the thrall of substance abuse disorders, compulsive behaviours and phobias may similarly make it more challenging to avoid criminal wrongdoing.

Although such factors do not, and ought not, constitute *defences* to crimes, many people share the intuition that they can be relevant to appropriate punishment. That concern becomes the basis of Erin Kelly’s theory of moral excuse, which she offers as a rival to an influential account proposed by PF Strawson and expounded further by R Jay Wallace, who think that a condition excuses a wrongdoer’s conduct precisely by showing that it did not manifest the kind or degree of bad ‘quality of will’ toward others (or, in the parlance of leading criminal law theorists, ‘insufficient concern’ for others) that such wrongdoing ordinarily manifests.^27^ Kelly, by contrast, writes: ‘One way to express [her] rationale for excuses is to say that it seems unfair that the requirements of morality are so much harder for some people to meet.’^28^ As Kelly appreciates, in the criminal law the category of ‘excuses’ of interest to her—ie those based on the special difficulty or costliness for some people, in some circumstances, of giving other people their due—would often be relevant only to mitigation at sentencing and not the adjudication of criminal guilt.^29^

What is important here is not how we use the terms ‘culpability’ and ‘excuse’, but that we recognise a crucial distinction—ie one between (i) the degree of ill will or deficiency of regard for others manifested in an offender’s conduct and (ii) whether and to what extent he faced special obstacles to avoiding manifesting the level of ill will or disregard for others’ interests that his criminal behaviour displayed. It is this distinction that makes it possible for an offender to be culpable in an important sense despite having lacked a fair opportunity to avoid becoming so.^30^ For it is possible to be culpable for a crime in the sense of having manifested the ill will or disregard for other people that makes the crime wrong, but also to have faced special (and potentially unfair) obstacles to avoiding manifesting one’s bad quality of will or inadequate concern for others. Not all difficulties or costs of avoiding wrongdoing mitigate the deficiency of regard for others’ rights and interests that such wrongdoing thereby manifests. Sometimes the problem is not that it can be difficult or costly even for well-intentioned agents to avoid wrongdoing, but, rather, that it can be difficult and costly for some people to avoid holding and acting on ill will or with genuinely insufficient concern for other people.^31^

## 4. Fair Opportunity in Employment and Punishment

I have referred to obstacles to manifesting adequate regard for other people as being only *potentially* unfair. Which ones are actually unfair, and why?

On the one hand, to appreciate that job qualifications are one thing and fair opportunity to develop them another, we do not need to settle difficult debates in the theory of distributive justice surrounding the meaning of substantively fair opportunity. One need not determine, for instance, whether strict equality of opportunity is required, or something less may suffice, to conclude that opportunities to develop job qualifications are subject to principles of distributive justice. Analogously, to make the case that it is possible to lack a fair opportunity to avoid incurring the criminal culpability that qualifies a person for punishment, it should not be necessary to articulate the precise principles by which opportunities to avoid criminal culpability must be allocated if everyone is to have a fair opportunity to avoid criminal culpability.^32^ On the other hand, whereas opportunities to develop job qualifications are already widely viewed as subject to principles of distributive justice, opportunities to avoid manifesting inadequate regard for other people are not yet widely viewed as subject to principles of distributive justice. Consequently, although it is not my burden to determine the precise content of the principles of fairness that govern the allocation of opportunities to avoid criminal culpability, I do owe readers at least some account of how poor opportunity to avoid criminal culpability can be unfair rather than merely unfortunate.

In the context of employment, it is easy to understand how a person might be unqualified for a job in part because she lacked a fair opportunity to develop the capacities, talents and skills that would enable her to perform the role well. For instance, because of childhood poverty, a job candidate may have had to attend poorly performing schools with high rates of behavioural problems among students, to endure the demoralisation and distraction of community violence, and to prepare for future employment with relatively little access to professional role models or mentors. Moreover, we need not endorse any particular, controversial views of social justice to be confident that when people face greatly varying obstacles to cultivating their capacities owing to circumstances of birth and childhood beyond their control, those individuals who face systematic developmental barriers plausibly lack a *substantively* fair opportunity to compete for jobs—even if the hiring processes are *formally* fair, in the sense that they allocate positions to the applicants who are most qualified for them.

Why, then, is it difficult to appreciate how a person might become qualified for punishment partly because he lacked a fair opportunity to foster the agential dispositions and environmental supports that would reliably protect him against resorting to crime? Part of the difficulty is that, as we saw in section 3, given the moral stigma that attaches to the criminal culpability that qualifies a person for punishment, and given that at least some (extreme) obstacles to avoiding *prima facie* criminal conduct are recognised as negating culpability, it is tempting to assume that for it to be appropriate for the criminal law to deem a person’s behaviour criminally culpable, it must be conduct that, in important senses, she had the capacity and fair opportunity to avoid. There is also a second, related reason it is difficult to imagine a person accruing qualification for punishment he lacked a fair opportunity to avoid: it is obvious how the ability to qualify for a job depends on human development, which in turn requires social support (eg socialisation, education and training), but it is less clear how the ability to avoid culpable crime depends similarly on human development requiring analogous social support. The path to qualification for sought-after employment is an uphill, competitive climb filled with obstacles and requiring assistance from others. By contrast, avoiding culpable criminal wrongdoing may appear comparatively easy to do on one’s own, without special opportunities or help from others.

It may appear to many readers that, for them anyway, there are not many serious hurdles to avoiding culpable crime. But that, I suspect, is because I am writing for a self-selected audience of comparatively privileged, well-socialised individuals who have, from an early age, had good opportunities to cultivate their habits of mind and body, and structure their choice environments, to minimise temptations and other pressures to resort to crime. It would be misleading to conceive of success in reliably avoiding crime as about continually resisting temptations and other pressures to violate other people’s rights or shortchange their interests. That would be akin to framing healthy living as winning in the constant struggle to motivate oneself to exercise and to resist urges to snack and overeat. To think about a healthy lifestyle in those terms would be—for those of us with only ordinary will power, anyway—a recipe for failure. So too would it be foolhardy to try to avoid crime by constantly making conscious choices to avoid it in the face of many pressures to engage in it.

As Jeffrey Howard has insightfully observed, it is plausible that we all have forward-looking moral *obligations* of what Howard calls ‘moral fortification’—ie duties to take preventive steps to help reduce the likelihood that we will succumb to any of the pressures that lead people to commit crimes.^33^ Moral fortification is partly about developing moral knowledge and cultivating a disposition to act in accordance with the demands of morality. But it is also a matter of entrenching oneself in environments that are low in temptations and other pressures to criminally offend. And we are not all equally well positioned to discharge our obligations of moral fortification. Inequalities in people’s life circumstances can greatly facilitate or obstruct moral fortification—whether *qua* personal moral development or *qua* orchestration of one’s environment to facilitate moral success. Indeed, those two issues are more separable analytically than in practice. For the kinds of habits that will be adaptive for a person to develop will depend upon the social conditions she inhabits.

Consider first, then, the difficulty of structuring one’s choice situations to avoid or minimise temptations and other pressures to commit crimes. What kinds of temptations and pressures lead people to offend?

Especially in affluent, highly unequal societies that promote materialism, avarice, competitiveness and envy, individuals experiencing poverty and lack of lawful economic opportunities will face special pressures to resort to economic crimes ranging from fraud and larceny to drug trafficking and to robbery and burglary. If they participate in illicit economic activity or perceive the legal system as illegitimate or ineffective in relation to them, they may resort to violence to resolve disputes. And they may perpetrate cycles of vigilante justice outside the law.^34^ If, as is often the case, their social circumstances make them more vulnerable to criminal victimisation, they may find it adaptive to protect themselves with pre-emptive threats (as part of a ‘code of the street’),^35^ leading to dispositions to use force beyond the limited contexts in which it would be legally justified as self-defence. There is also important social science research demonstrating that poverty in an affluent society strains people’s cognitive and volitional capacities by preoccupying them with making ends meet and avoiding temptations to overspend.^36^ And a criminal record and prison term can themselves be criminogenic, partly because of the broad range of formal and informal collateral consequences that flow from them, including diminished labour market opportunities.^37^

Members of oppressed groups may also be more likely to engage in crime—including violent crime—as a result of intergenerational trauma stemming from deep and long-standing historical injustice.^38^ Personal, familial and widespread community traumas may manifest themselves in alienation, anger and problems with emotional and behavioural regulation, which may in turn trigger such harmful coping mechanisms as self-medication through the abuse of alcohol or other drugs, or lateral violence (ie repressed anger at one’s oppression that is redirected toward friends, family or others in the oppressed group of which one is a member). And such consequences of trauma in parents can negatively impact their children in innumerable ways—whether by causing foetal alcohol spectrum disorders or other pregnancy-related harms, or by increasing the risk of familial instability, abuse or neglect. Moreover, because anger and alienation are frequently *appropriate* responses to social oppression, marginalisation and exclusion, members of groups that have been the victims of historical injustice and intergenerational trauma may face unfair burdens managing emotions that, though often righteous, are fraught with the potential to spill over into deviant, antisocial and self-sabotaging behaviour.^39^ Amia Srinivasan has eloquently characterised this as ‘*affective* injustice: the injustice of having to negotiate between one’s apt emotional response to the injustice of one’s situation and one’s desire to better one’s situation’.^40^

The problem is not merely that people from unjustly disadvantaged backgrounds are likely to encounter more frequent and severe constraints on opportunities as well as temptations and other pressures to commit crimes (though that certainly appears to be one important problem^41^). Although the most salient difficulties and costs of avoiding crime are those that are contemporaneous with wrongdoing (eg immediate threats, temptations and psychological pressures), if we bear in mind both the importance of moral fortification and our guiding analogy between fair opportunity to qualify for employment and fair opportunity to *avoid* qualifying for punishment, we cannot help but begin to recognise the underappreciated importance of the *aetiology* of whatever factors made it difficult and/or costly for an offender to avoid his crime. As has sometimes been acknowledged explicitly, the weight we accord to difficulties or costs of avoiding crime ought to depend upon facts related to how the offender came to face them—in particular, whether, and to what extent, the agent had a fair opportunity to fortify herself against them.^42^

For instance, if a drug addict commits a property offence, whether his addiction deprived him of a fair opportunity to avoid his crime would appear to depend on whether he had a fair opportunity to avoid the addiction in the first place. If a young man has committed a crime of hot-tempered aggression, we should like to know: how conducive were his background circumstances in life to avoiding the development of a bad temper and to cultivating strategies for deescalating it? If, instead, he has sold drugs or burgled a home because of peer pressure, we should consider whether the circumstances of his upbringing accorded him fair alternative economic opportunities and a fair opportunity to avoid falling prey to others’ coercive influence.

Contemporaneous difficulties or costs of avoiding an offence may not be *sufficient* grounds for mitigating punishment if an offender had a fair opportunity to avoid them. What is more, contemporaneous difficulties or costs of avoiding an offence may not be *necessary* for an offender to have lacked a fair opportunity to avoid his crime. That is so for two reasons.

First, irrespective of whether an offender suffered from some form of ‘diminished capacity’ when he committed his crime, there is a further question of whether he had a fair opportunity for the moral and agential development, across his life, that would position him well to give others their due in each situation in which he should find himself. For instance, if we compare two different offenders with equally underdeveloped powers of moral reflection and self-control, one of whom is an adolescent and the other of whom is an adult, our greater sympathy for the adolescent may partly reflect the sense that the adolescent has not yet had the same opportunities for moral and agential development as his adult counterpart, whose development has remained arrested despite more opportunities for it to continue.^43^

Second, irrespective of whether she suffered from some immediate pressure to offend, there is a further question of whether an offender had a fair opportunity to structure her choice environment to minimise pressures to offend and maximise safeguards against succumbing to crime. Even when there was no dramatic difficulty or cost of avoiding an offence in the moment, it might still be the case that the offender could have protected herself against succumbing to crime through prior choices that would restrict her options to offend, reinforce her disposition against offending, and thereby give her insurance against a lapse of judgment or self-control. Yet she might also have lacked a fair opportunity to fortify herself in that way because of her disadvantaged background.

The idea that a criminal offender’s lack of fair opportunity for moral fortification undermines the fairness of his opportunity to avoid punishment differs in kind from the idea that the fairness of punishment is undermined by the strict or probabilistic determination of offenders’ conduct by forces beyond their control.^44^ If it were to turn out that all human behaviour is ultimately determined strictly or probabilistically by factors outside each agent’s control, the very universality of such a truth would prevent it from explaining why we do or should draw distinctions among different wrongdoers and say that some are excused and others are not, and some should have their punishment mitigated and others should not.^45^ Just as we recognise that certain immediate forces beyond our control (eg threats of death or bodily harm) are different from the garden-variety factors that shape us all, so too should we acknowledge that certain historical, background life circumstances beyond our control (ie those that generate a lack of fair opportunity for moral fortification) are different from the innumerable, ordinary determinants of human behaviour that are neither fair nor unfair.

Sceptics of the relevance of a disadvantaged background to the fairness of punishment may nevertheless argue that there is no principled moral difference between those who lack moral fortification owing to a history of abuse, deprivation or oppression and those who lack moral fortification because, like Ethan Couch, they have suffered from ‘affluenza’ or been ‘spoiled rotten’ by privilege.^46^ Some critics may go even further and argue that social *advantage* is morally compromising (perhaps because power corrupts) and that social disadvantage ennobles people by directly attuning them to injustice and its consequences. But I suspect that such claims rest on ambiguity and misunderstanding surrounding the idea of moral fortification. For the purposes of criminal responsibility and punishment, the kind of moral fortification we should be concerned about is fortification against engaging in culpable crime. Even if it should turn out that social advantage is associated with various moral vices, and social disadvantage is a pathway to certain moral virtues, we have strong reason to believe that social disadvantage amid affluence and inequality is criminogenic.^47^ Moreover, social disadvantage may appear ennobling and social advantage morally compromising because, outside the criminal law, we sometimes conceive of moral success and failure as relative to the adversity of one’s circumstances, such that the privileged must do more than the disadvantaged to be true moral successes. Yet such a perspective presupposes precisely that social advantage *is* conducive to the kind of surface level moral success—not relativised to people’s life circumstances—that is the primary concern of the criminal law.

## 5. *A Case Study: The* Gladue *Principles*

Where fair opportunity for moral fortification does not exist, we have reason to bring it about. But altering society to do so may not be any easier than realising fair economic opportunity in a substantive sense. If we are unable to realise either form of fair opportunity anytime soon, a potential second-best alternative that may be more immediately accessible is altering the process for allocating jobs or punishment, to give preferential treatment to those who lacked fair substantive opportunity to qualify for employment or to avoid qualifying for punishment. This is the line of thinking that should lead us to consider affirmative action in criminal justice—that is, mitigating the punishment of any given culpable crime when and because the perpetrator’s background circumstances in life were such that he did not have a fair opportunity to cultivate the agential capacities and dispositions, and the social and environmental supports, that give people reliable security against resorting to crime.

Arguably, the special *Gladue* principles that apply to the sentencing of Indigenous offenders in Canada can be profitably understood as—however partially and inchoately—grasping at the idea of affirmative action in criminal justice of precisely the sort imagined in this article.^48^ In 1995, in response to concern about the level of incarceration in Canada—especially of Indigenous people, who were (and remain) conspicuously overrepresented in prison—Parliament passed legislation creating section 718.2(*e*) of Canada’s Criminal Code, which went into force the following year and stated:

A court that imposes a sentence shall also take into consideration the following principles … (*e*) all available sanctions other than imprisonment that are reasonable in the circumstances should be considered for all offenders, with particular attention to the circumstances of Aboriginal offenders.^49^

In the landmark case of *R v Gladue* (1999), the Supreme Court of Canada held that the provision was intended to remedy the problem of overincarceration in general, and the overincarceration of Indigenous people in particular.^50^ But it does not call for a crude or automatic reduction in the incidence and duration of custodial sentences for Indigenous people.^51^ Instead, to quote the Court, in sentencing Indigenous offenders, judges are to bring two key considerations to bear on their application of the principle of restraint:

(A) The unique systemic or background factors which may have played a part in bringing the particular aboriginal offender before the courts; and
(B) The types of sentencing procedures and sanctions which may be appropriate in the circumstances for the offender because of his or her particular aboriginal heritage or connection.^52^

My suggestion is that the notion of affirmative action in criminal justice explicated in this article may provide a useful framework for understanding how and why sentencing judges should attend to (A).^53^ In *Gladue*, the Supreme Court opined:

The background factors which figure prominently in the causation of crime by aboriginal offenders are by now well known. Years of dislocation and economic development have translated, for many aboriginal peoples, into low incomes, high unemployment, lack of opportunities and options, lack or irrelevance of education, substance abuse, loneliness, and community fragmentation.^54^

In its underdeveloped initial effort to explain why those background factors are relevant to a fit sentence, the Supreme Court in *Gladue* suggested that insofar as background social disadvantages of those kinds may help to explain an Indigenous offender’s crime, a custodial sentence may fail to deter or communicate condemnation in a way that is meaningful to the offender.^55^ Later, in *R v Ipeelee* (2012), the Supreme Court emphasised the need for judges to consider ‘constrained circumstances’ that mitigate the offender’s blameworthiness or ‘degree of responsibility’ such that proportionality calls for a reduction in punishment.^56^

The Supreme Court’s clear hope has been that if judges consistently and appropriately consider the factors to which *Gladue* requires them to attend, this should reduce the incarceration of Indigenous offenders overall by leading judges to do a better job, respectively, of (i) recognising the unique background factors in Indigenous offenders’ lives that may have mitigating force at sentencing and (ii) avoiding ineffective or counterproductive custodial sentences. Unfortunately, the *Gladue* principles have not yet had their intended remedial impact. Indigenous overrepresentation in prison remains dramatic and appears to have grown in the 21st century.^57^ Part of the problem, however, appears to be inadequate application of *Gladue* in practice, as has been identified in various doctrinal and empirical studies.^58^ In *Ipeelee*, looking back on over a decade of experience with the *Gladue* principles, the Supreme Court of Canada lamented:

s. 718.2(*e*) of the *Criminal Code* has not had a discernible impact on the overrepresentation of Aboriginal people in the criminal justice system. Granted, the *Gladue* principles were never expected to provide a panacea. There is some indication, however, from both the academic commentary and the jurisprudence, that the failure can be attributed to some extent to a fundamental misunderstanding and misapplication of both s. 718.2(*e*) and this Court’s decision in *Gladue*.^59^

The Court went on to ‘attempt to resolve these misunderstandings, clarify certain ambiguities, and provide additional guidance so that courts can properly implement this sentencing provision’.^60^ It did so not only by emphasising that ‘constrained circumstances’ can be mitigating, but also by stressing that the *Gladue* principles apply in the sentencing of all Indigenous offenders; unfortunately, judges had sometimes wrongly assumed those principles to be inapplicable or unimportant merely because an offender committed a ‘serious’ crime or could not trace a clear causal connection between her life circumstances and that crime.^61^ However, the authors of a study of 635 decisions post-dating *Ipeelee* involving the sentencing of Indigenous offenders found that in the vast majority of cases judges still either did not mention ‘background and systemic factors’, minimised their significance or purported to take them seriously but without clearly explaining their impact.^62^ The implementation of *Gladue* has been checkered at best.

Yet, if sentencing judges have often struggled to analyse and articulate *Gladue* factors in a clear and robust way, rather than in a vague and perfunctory one, that shows the *need* for a rigorous theory of something like ‘affirmative action in criminal justice’ and not that implementing such a theory would be infeasible in principle. Moreover, *Gladue* presents a theoretical conundrum that the concept of affirmative action in criminal justice helps us to resolve in an analytically tidy and (arguably) normatively attractive way.^63^

Unsurprisingly, the Supreme Court wants to have it both ways by presenting the *Gladue* principles as distinctive to the sentencing of Indigenous offenders (and capable of having a remedial impact on Indigenous overincarceration) yet also merely a particular instantiation of the fundamental principle of Canadian sentencing: ‘A sentence must be proportionate to the gravity of the offence and the degree of responsibility of the offender.’^64^ Section 718.2(*e*) of the Criminal Code enjoins judges to consider reasonable alternatives to incarceration for all offenders, albeit ‘with particular attention to the circumstances of Aboriginal offenders’. The Supreme Court has thus repeatedly clarified that ‘background and systemic factors will also be of importance for a judge in sentencing a non-aboriginal offender’.^65^ Distinctive systemic or background factors that help to explain why a specific individual finds herself being sentenced by a criminal court should be considered in all cases, but section 718.2(*e*) reminds judges of the special likelihood of such factors being relevant in the case of Indigenous offenders, given the unique historical injustice and intergenerational trauma that Indigenous peoples of Canada have faced as a result of such settler–colonialist policies as displacement from historic lands, the residential school system, and associated practices of forced family separation and cultural assimilation.

It is tempting, therefore, to construe component (A) of the *Gladue* analysis as merely a reminder to judges to do what they would anyway be required to do: to be vigilant against implicit biases and to undertake a careful, individualised assessment of how, if at all, each offender’s background circumstances may bear upon a fit sentence.^66^ Yet, although the Indigenous overrepresentation in prison that *Gladue* seeks to reduce may partly reflect invidious discrimination at sentencing, the Supreme Court itself has acknowledged that higher levels of criminal offending appear also to be an important source of disparities in outcomes.^67^ And if the *Gladue* principles were merely a means of ensuring that judges check their potential biases and carefully apply the ordinary principles of sentencing to Indigenous offenders, then they would not add to the content of the law but would serve only as a redundant exhortation to continue to apply it dispassionately and steadfastly where Indigenous offenders are concerned.^68^

By contrast, if *Gladue* were understood as calling for affirmative action for offenders who have lacked fair opportunity for moral fortification, then it could be seen as widening the scope of sentence mitigation in a way that might meaningfully combat Indigenous overincarceration. Interpreted as affirmative action in service of substantive fairness, *Gladue* would invite judges to look beyond the immediate circumstances of an offender’s crime (such as whether he faced a discrete ‘hard choice’) and to consider whether, across his life—and in relation to the overall distribution of opportunities in his society—it could be said that the offender had a fair opportunity to develop personal and environmental safeguards and supports that would help him reliably to avoid succumbing to crime.^69^

Earlier in this article I showed that it is important not only to distinguish between (i) the degree of disregard for others manifested in a crime and (ii) the difficulty or costliness of avoiding such a degree of disregard, but also to consider whether the offender had a fair opportunity to fortify himself against pressures to offend—both by cultivating his inner capacities and dispositions, and by structuring his choice environments so as to minimise pressures to offend and maximise protections against succumbing to them. On the one hand, sometimes immediate pressures should be written off or downplayed precisely because the offender had a fair opportunity to avoid them by making better prior choices. On the other hand, however strong or weak were the immediate pressures to offend, constraints on a wrongdoer’s opportunities in life may have unfairly limited her ability to fortify herself against those pressures. The Supreme Court of Canada seems to have been grasping for that point (though not quite reaching it) when it wrote in *Gladue*:

It is true that systemic and background factors explain in part the incidence of crime and recidivism for non-aboriginal offenders as well. However, it must be recognized that *the circumstances of aboriginal offenders differ from those of the majority* because many aboriginal people are victims of systemic and direct discrimination, many suffer the legacy of dislocation, and many are substantially affected by poor social and economic conditions.^70^

What the Supreme Court here comes close to recognising is that any given immediate pressures to offend may be of differential significance for sentencing depending upon their origins and the circumstances in which they arose. Only when we have considered the broader range of obstacles and opportunities relevant to an offender’s fortification against resorting to crime can we be sure that the offender had a substantively fair opportunity to avoid his offence. This is true for Indigenous and non-Indigenous offenders alike. Indigenous people’s unique history of mistreatment and their overrepresentation in prison offer an especially vivid illustration of a general lesson about how penal fairness may depend on offenders’ life stories and historical injustices in which they are entangled.

## 6. Conclusion

A hiring process may be formally fair because the employer selects candidates impartially for their job qualifications, yet nevertheless substantively unfair because some job candidates have been deprived of a fair opportunity to develop the capacities and skills to make them competitive with other candidates. Likewise, a criminal justice system might be formally fair because it imposes punishment only on the guilty and in proportion to their culpability, yet substantively unfair because some offenders have lacked a fair opportunity in life to cultivate their agential dispositions and shape their choice environments in ways that would reliably fortify them against succumbing to crime.

One familiar way to implement affirmative action in hiring is to accord preferential treatment to job candidates who lacked fair opportunities to develop their qualifications, to help the hiring process go beyond formal fairness and achieve a greater measure of substantive fairness. An analogous form of affirmative action in criminal justice would involve mitigating the punishment of offenders who lacked a fair opportunity to avoid their criminal culpability—ie their criminal manifestation of insufficient regard for other people’s rights and interests.

Canada’s *Gladue* principles for the sentencing of Indigenous offenders offer a concrete illustration of how affirmative action in criminal justice might be implemented in practice. To see what affirmative action in criminal justice might look like in a more general form, we may simply imagine a revised version of section 718.2(*e*) of the Criminal Code and *Gladue* focused not only on Indigenous offenders specifically, but on all those offenders ‘who belong to a vulnerable population that is overrepresented in the criminal justice system and that is [unfairly] disadvantaged in [avoiding incarceration]’.^71^ Membership in such a group could trigger judicial scrutiny of an offender’s background to determine whether he, specifically, was deprived of fair opportunity to fortify himself against the kind of criminal offending in which he engaged.

Even when it is formulated in a race-neutral manner and focused on substantive fairness rather than other rationales such as diversity, affirmative action is bound to remain controversial. One obvious reason is that many more people recognise a difference between formal and substantive fairness than agree on the content of substantive fairness. Affirmative action in criminal justice might vest a degree of discretion in judges that some sentencing philosophies would condemn. And there are other possible objections. In addition to holding the potential to compromise employers’ aims, affirmative action in hiring for the sake of substantive fairness risks harming those it is intended to help—for example, by stigmatising their achievements or weakening their incentive to work hard. Analogously, affirmative action in criminal justice would hardly commend itself if it were to involve penal leniency so significant as to increase the level of offending by members of disadvantaged groups (which would be bad both for them and for other members of their communities). Relatedly, affirmative action in the criminal law might be thought to impose a patronising ‘double standard’ that would stigmatise its intended beneficiaries. Finally, although affirmative action is most often criticised from the right side of the political spectrum, leftists also sometimes critique it as an ameliorative measure the benefits of which may be outweighed by its tendency to provoke conservative backlash while simultaneously shoring up the legitimacy of institutions requiring more fundamental overhauls.

But I do not purport to have offered a comprehensive defence of affirmative action in criminal justice even in theory—let alone to have addressed the many challenges of institutional design that would be involved in implementing it in practice. What I have tried to do is to give a theoretical foundation to an overlooked explanatory paradigm for thinking through the problem posed by the disparate punishment borne by the socially disadvantaged. Mainly, I hope simply to have expanded readers’ understanding of what is wrong with the concentration of punishment on some of the most disadvantaged members of society.

## Footnotes

1 The Indigenous percentages of federal prisoners and of the total Canadian population noted in the text come from the following sources: Statistics Canada, ‘Indigenous Population Continues to Grow and Is Much Younger Than the Non-Indigenous Population, Although the Pace of Growth Has Slowed’ (Statistics Canada, 21 September 2022) Catalogue No 11-001-X, 2 <www150.statcan.gc.ca/n1/daily-quotidien/220921/dq220921a-eng.pdf> accessed 22 October 2024; Office of the Correctional Investigator, ‘Proportion of Indigenous Women in Federal Custody Nears 50%’ (Office of the Correctional Investigator, 17 December 2021) <https://oci-bec.gc.ca/en/content/proportion-indigenous-women-federal-custody-nears-50> accessed 22 October 2024.

2 Wendy Sawyer and Peter Wagner, ‘Mass Incarceration: The Whole Pie 2025’ (Prison Policy Initiative, 11 March 2025) <https://www.prisonpolicy.org/reports/pie2025.html> accessed 9 April 2025.

3 See eg Nathaniel Lewis, ‘Mass Incarceration: New Jim Crow, Class War, or Both?’ (People’s Policy Project 2018) <www.peoplespolicyproject.org/wp-content/uploads/2018/01/MassIncarcerationPaper.pdf> accessed 22 October 2024; Scott Clark, ‘Overrepresentation of Indigenous People in the Canadian Criminal Justice System: Causes and Responses’ (Department of Justice Canada 2019) 15–17 <www.justice.gc.ca/eng/rp-pr/jr/oip-cjs/oip-cjs-en.pdf> accessed 22 October 2024.

4 For instance, early in the present century Bruce Western, the leading sociological critic of mass incarceration in America, judged that ‘High rates of homicide among black men fully explain the parallel high rates of imprisonment for murder’ and noted that ‘Criminologists estimate that about 80 percent of black-white difference in imprisonment rates is due simply to the high involvement of black men in crime’. Bruce Western, *Punishment and Inequality in America* (Russell Sage Foundation 2006) 50. More recently, Western has noted: ‘Researchers distinguish between the “warranted” racial disparities that are attributable to crime and the “unwarranted disparities” that remain after crime rates have been taken into account. These studies show that more than half the racial disparity in imprisonment is “warranted” in this sense.’ Bruce Western, *Homeward: Life in the Year After Prison* (Russell Sage Foundation 2018) 171. There is also evidence that in Canada Indigenous overrepresentation among the perpetrators of serious crime has played a substantial role in explaining Indigenous overrepresentation in prison. For instance, in 2015, despite being only about 5% of the population, Indigenous people constituted about a quarter of homicide victims in Canada. Indigenous people also made up ‘90% of accused implicated in the homicides of Indigenous victims’ and ‘33% of people accused of homicide’. Research and Statistics Division, Department of Justice Canada, ‘JustFacts: Indigenous Overrepresentation in the Criminal Justice System’ (Department of Justice Canada, January 2017) 1–2 <www.justice.gc.ca/eng/rp-pr/jr/jf-pf/2017/docs/jan02.pdf> accessed 22 October 2024.

5 See eg Christopher Lewis and Adaner Usmani, ‘The Injustice of Under-Policing in America’ (2022) 2 American Journal of Law and Equality 85, 88.

6 For a concise overview of the distinction between ‘formal’ and ‘substantive’ equality of opportunity, see Richard Arneson, ‘Equality of Opportunity’, *The Stanford Encyclopedia of Philosophy* (Summer edn, 2015) <plato.stanford.edu/archives/sum2015/entries/equal-opportunity/> accessed 22 October 2024. See also TM Scanlon, *Why Does Inequality Matter?* (OUP 2018) 53–73; Bernard Williams, ‘The Idea of Equality’ in Geoffrey Hawthorn (ed), *In the Beginning Was the Deed: Realism and Moralism in Political Argument* (Princeton UP 2005) 109–12; Charles Frankel, ‘Equality of Opportunity’ (1971) 81 Ethics 191, 202–4.

7 For a limited defence of a policy of this sort to compensate people for unfair competitive disadvantages, see George Sher, ‘Justifying Reverse Discrimination in Employment’ (1975) 4 Philosophy & Public Affairs 159.

8 As will become clear as the article unfolds, the idea of ‘affirmative action in criminal justice’ of interest to me is neither an essentially race-based notion nor a policy aimed at promoting diversity—unlike the type of ‘affirmative action’ the Supreme Court of the United States recently struck down in college admissions. See *Students for Fair Admissions, Inc v President & Fellows of Harvard Coll* 600 US 181 (2023).

9 To the best of my knowledge, since the emergence of the concept of ‘affirmative action’ in the United States in the 1960s, there have been only a few scattered scholarly discussions of the possibility of affirmative action in criminal justice (none of which has grounded the idea in a sustained comparison of formal and substantive fairness in employment and punishment). See especially Bruce P Archibald, ‘Sentencing and Visible Minorities: Equality and Affirmative Action in the Criminal Justice System’ (1989) 12 Dalhousie Law Journal 377; Paul Butler, ‘Affirmative Action and the Criminal Law’ (1997) 68 U Colo L Rev 841; Jacob D Humerick, ‘Reprogramming Fairness: Affirmative Action in Algorithmic Criminal Sentencing’ (2020) 4 Colum Hum Rts L Rev Online 213. Butler’s article, the lengthiest treatment of the issue, called for affirmative action in criminal justice in the sense of race-based preferential treatment of Black people in America’s criminal justice systems, justified by appeal to a range of traditional grounds for race-based affirmative action in other contexts. Archibald’s brief discussion called for affirmative action at sentencing as a way of going beyond formal equality in sentencing to correct for ‘systemic discrimination’ that produces disproportionately high incarceration rates for disadvantaged groups. See Archibald (n 9) 403–8. However, Archibald appears to have conceived the justification of his proposal in terms of the supposedly lesser culpability of the policy’s beneficiaries, which is not my approach. See ibid 406. Finally, Humerick has recently argued that Black Americans should be given credit in their favour in recidivism risk assessments. But his proposal appears focused on screening out racial bias in such assessments, rather than compensating for unfairly criminogenic conditions.

10 It is also worth noting that this article’s answer to the question of what might be wrong with unmitigated punishment of socially disadvantaged offenders can and should be assessed on its own terms. It does not preclude the possibility of alternative diagnoses, such as the idea that unjust states lack the authority or moral ‘standing’ to blame the victims of their injustice. Elsewhere, I have discussed at length unjust states’ standing to blame disadvantaged offenders, and other approaches to the problem of criminal justice for the unjustly disadvantaged. See Benjamin Ewing, ‘Do Unjust States Have the Standing to Blame: Three Reservations About Scepticism’ (2023) 43 OJLS 249; Benjamin Ewing, ‘Recent Work on Punishment and Criminogenic Disadvantage’ (2018) 37 Law and Philosophy 29.

11 HLA Hart, *Punishment and Responsibility: Essays in the Philosophy of Law* (John Gardner ed, 2nd edn, OUP 2008) 3–13.

12 See eg Michael S Moore, ‘Justifying Retributivism’ (1993) 27 Israel Law Review 15, 48.

13 Scanlon, *Why Does Inequality Matter?* (n 6) 70.

14 In this article I abstract from the distinction between public and private employers. Arguably, private employers should have greater leeway in their hiring decisions than public ones.

15 Lippert-Rasmussen has developed a useful typology of six possible justifications for affirmative action. The other justifications are compensating for historical injustice against disadvantaged groups, combating present discrimination against them, increasing the number of their members in desirable positions who can then serve as role models, promoting diversity and its associated values, and integrating disadvantaged groups into the broader society. See Kasper Lippert-Rasmussen, *Making Sense of Affirmative Action* (OUP 2020).

16 I focus on criminal sentencing, rather than guilt or offence of conviction, because affirmative action in criminal justice, as I conceive of it, is not grounded in even partial justification or excuse, but in the theory that affirmative action’s beneficiaries lacked a fair opportunity to avoid the extent of their culpability and thus the punishment it would ordinarily license. I focus on hiring because it is the most salient site of affirmative action in the context of employment. As an anonymous reviewer for this journal has noted, affirmative action efforts have focused on decisions about hiring rather than termination. And if the potential stigma of being fired justifies a reluctance to undertake affirmative action in decisions about termination (eg by treating an employee’s lack of fair background opportunity as a reason to prioritise his job retention over that of other employees), this might undercut the justification of affirmative action in punishment, which stigmatises much more than being laid off from work. In s 3, I respond to the objection that the distinctive stigma of punishment, which is not at stake in decisions about hiring, might trouble the case for affirmative action in criminal justice. Yet it is not clear to me that it would be inappropriate to treat an employee’s lack of fair opportunity to develop her job qualifications as one reason to cut her slack in performance assessment and to be slower to resort to firing her—whether for reasons of cost-cutting necessity or deficient performance. And quite apart from the issue of stigma, there are good reasons to undertake affirmative action first with respect to hiring and second, if at all, with respect to retention. For starters, we cannot safeguard the employment of individuals who lacked fair background opportunities to develop their job qualifications unless and until they have secured that employment in the first place. And if we have already undertaken affirmative action in hiring, in undertaking further affirmative action in retention we need to take care to avoid moral double-counting of people’s unfairly diminished developmental opportunities.

17 It is frequently argued that traditional measures of ‘qualification’ are biased against disadvantaged groups. See eg Joseph Fishkin, *Bottlenecks: A New Theory of Equal Opportunity* (OUP 2014) 32–5.

18 cf Butler (n 9) 868–9 (describing a conception of ‘affirmative action [in criminal law that] critiques the traditional construct of demerit in the criminal context’ and seeks ‘to reconstruct the criteria by which criminals are selected to make those criteria more efficient and fair’). Various legal philosophers have proposed new partial excuses or mitigating factors they believe would apply disproportionately to offenders from disadvantaged backgrounds. Tommie Shelby, for instance, has suggested that the Black urban poor in America may be justified or excused when committing certain economic crimes, such as selling drugs, if the wrongfulness or culpability of those offences depends on them being violations of civic reciprocity. See Tommie Shelby, ‘Justice, Deviance, and the Dark Ghetto’ (2007) 35 Philosophy & Public Affairs 126. Richard Lippke has suggested that the excuse of duress should be supplemented with a recognition of the partially exculpatory force of chronic temptation—a condition especially likely to have affected offenders from disadvantaged backgrounds. See Richard L Lippke, ‘Chronic Temptation, Reasonable Firmness and the Criminal Law’ (2014) 34 OJLS 75, 76. Finally, Christopher Lewis has argued that (i) we have less evidence of crime perpetrators’ culpability when they have acted in the face of objectively greater incentives to do wrong and (ii) disadvantaged offenders tend to have objectively greater incentives to offend. See Christopher Lewis, ‘Inequality, Incentives, Criminality, and Blame’ (2016) 22 Legal Theory 153, 161–70.

19 See eg David O Brink, ‘Partial Responsibility and Excuse’ in Heidi M Hurd (ed), *Moral Puzzles and Legal Perplexities: Essays on the Influence of Larry Alexander* (CUP 2018); Douglas N Husak, ‘Partial Defenses’ (1998) 11 CJLJ 167.

20 The need for punishment to target conduct that ought to be discouraged and condemned may be straightforwardly derived from the very concept of punishment, which is widely accepted to entail both hard treatment and condemnation. See Joel Feinberg, ‘The Expressive Function of Punishment’ in *Doing and Deserving: Essays in the Theory of Responsibility* (Princeton UP 1970).

21 See especially Larry Alexander and Kimberly Kessler Ferzan (with Stephen J Morse), *Crime and Culpability: A Theory of Criminal Law* (CUP 2009); Larry Alexander, ‘Insufficient Concern: A Unified Conception of Criminal Culpability’ (2000) 88 CLR 931. See also Alexander Sarch, ‘Who Cares What You Think? Criminal Culpability and the Irrelevance of Unmanifested Mental States’ (2017) 36 Law and Philosophy 707; Peter Westen, ‘An Attitudinal Theory of Excuse’ (2006) 25 Law and Philosophy 289.

22 Gideon Yaffe, *The Age of Culpability: Children and the Nature of Criminal Responsibility* (OUP 2018) 66–97, 125–57.

23 Yaffe’s theory of criminal culpability is more general than that of Alexander and Ferzan (n 21), in that the former leaves open the possibility that the wrong-making features of a crime may, from the criminal law’s point of view, include facts about it beyond its impact on the rights and interests of other people. See Yaffe (n 22) 77–9. For simplicity and ease of exposition, however, I will focus on culpability *qua* manifestation of insufficient concern for others.

24 See especially Alexander and Ferzan (n 21) 25–31; Alexander (n 21) 933–5.

25 Though duress is often thought merely to excuse, some have argued that it justifies would-be criminal wrongdoing. See Peter Westen and James Mangiafico, ‘The Criminal Defense of Duress: A Justification, Not an Excuse—And Why It Matters’ (2003) 6 Buffalo Criminal Law Review 833.

26 See eg Alexander and Ferzan (n 21) 156; Westen (n 21) 364–7.

27 Erin Kelly, ‘What Is an Excuse?’ in D Justin Coates and Neal A Tognazzini (eds), *Blame: Its Nature and Norms* (OUP 2013) 247. cf Peter Strawson, ‘Freedom and Resentment’ in Gary Watson (ed), *Free Will* (2d edn, OUP 2003); R Jay Wallace, *Responsibility and the Moral Sentiments* (Harvard UP 1994) 118–53.

28 Kelly (n 27) 256.

29 ibid 249.

30 In contrasting obstacles to avoiding wrongdoing that do and do not diminish the ill will or insufficient concern for others manifested in that wrongdoing, and in showing how the distinction between them makes it possible to be disadvantaged in one’s ability to avoid the ‘culpability’ that qualifies a person for punishment, I am simplifying a previous analysis of this distinction in which I framed it as the difference between (i) obstacles to avoiding wrongdoing that ‘weaken the moral reasons to avoid it’ and (ii) obstacles to ‘acting in accordance with moral reason’. See Benjamin Ewing, ‘Criminal Responsibility and Fair Moral Opportunity’ (2023) 17 Criminal Law and Philosophy 291.

31 When I mention difficulty and costliness, I invoke GA Cohen’s understanding of the former as about the low odds or unlikelihood of accomplishing something and the latter as about burdens or setbacks to one’s interests it will require. See GA Cohen, ‘On the Currency of Egalitarian Justice’ (1989) 99 Ethics 906, 918–19.

32 Hence, although I have elsewhere offered a tentative sketch of principles of fairness in people’s ‘moral opportunities’ by which to avoid culpable crime (see Ewing, ‘Criminal Responsibility and Fair Moral Opportunity’ (n 30)), and the present discussion draws and builds on that previous work, my reflections here are less ambitious. They are focused on illustrating that people’s background opportunities in life pervasively shape their ability to fortify themselves against criminal wrongdoing much like their ability to develop qualifications for employment.

33 Jeffrey W Howard, ‘Punishment as Moral Fortification’ (2017) 36 Law and Philosophy 45. See also Youngjae Lee, ‘Recidivism as Omission: A Relational Account’ (2009) 87 Tex L Rev 571 (also recognising that agents have forward-looking obligations to take preventive steps to minimise the likelihood they will commit crimes).

34 See eg Jill Leovy, *Ghettoside: A True Story of Murder in America* (Spiegel & Grau 2015).

35 See eg Elijah Anderson, *Code of the Street: Decency, Violence, and the Moral Life of the Inner City* (WW Norton and Co 1999).

36 See Sendhil Mullainathan and Eldar Shafir, *Scarcity: Why Having Too Little Means So Much* (Times Books 2013).

37 For analysis of the adverse effects of a criminal record and imprisonment on expected future employment and earnings, see Devah Pager, *Marked: Race, Crime, and Finding Work in an Era of Mass Incarceration* (University of Chicago Press 2007); Western, *Punishment and Inequality in America* (n 4) 108–30.

38 For discussion of intergenerational trauma among Indigenous peoples of Canada, see eg William Aguiar and Regine Halseth, ‘Aboriginal Peoples and Historic Trauma: The Processes of Intergenerational Transmission’ (National Collaborating Centre for Aboriginal Health 2015) <www.ccnsa-nccah.ca/docs/context/RPT-HistoricTrauma-IntergenTransmission-Aguiar-Halseth-EN.pdf> accessed 22 October 2024; Cynthia C Wesley-Esquimaux and Magdalena Smolewski, *Historic Trauma and Aboriginal Healing* (Aboriginal Healing Foundation 2004).

39 Amia Srinivasan has emphasised that anger is an ‘apt’ response to injustice even when counterproductive, which is also to say that it can be counterproductive even when it is apt. See Amia Srinivasan, ‘The Aptness of Anger’ (2018) 26 Journal of Political Philosophy 123.

40 ibid 135.

41 See eg Lippke (n 18); Lewis, ‘Inequality, Incentives, Criminality, and Blame’ (n 18) 161–70.

42 See Kelly (n 27) 259.

43 For further discussion, see Benjamin Ewing, ‘Youth as Moral Opportunity’ Legal Theory (forthcoming).

44 This point comes through strongly, though not in precisely the same terms, in TM Scanlon, *What We Owe to Each Other* (Harvard UP 1998) 248–94.

45 This is a point made famously and eloquently by Strawson (n 27).

46 See Lisa Maria Garza and Timothy Williams, ‘Teenager in “Affluenza” Case Is Sentenced to Jail’ *New York Times* (14 April 2016) A11.

47 See eg Lewis and Usmani (n 5) 88.

48 For a more detailed and doctrinal normative reconstruction of *Gladue* than I can offer here, which is consistent with—but does not entertain or discuss—the idea that *Gladue* might be, in part, a form of affirmative action in criminal justice analogous to affirmative action in employment, see Benjamin Ewing and Lisa Kerr, ‘Reconstructing *Gladue*’ (2024) 74 UTLJ 156.

49 Criminal Code, RSC 1985, c C-46.

50 [1999] 1 SCR 688, paras 24–65.

51 See ibid paras 88, 93; *R v Ipeelee* 2012 SCC 13, paras 71, 75.

52
*Gladue* (n 50) paras 66, 93. Since *Gladue*, Indigenous offenders have often submitted information about their Indigenous heritage or connection (and its relevance to the application of *Gladue*) in the form of special presentencing reports that have come to be called ‘*Gladue* reports’. See *Ipeelee* (n 51) para 60.

53 Although reflection upon (B) is an important part of what makes the *Gladue* principles distinctive, the rationale for incorporating it into the determination of a fit sentence is different in kind from the justification of the sort of ‘affirmative action’ sentencing mitigation of interest to me here. It is important to consider (B), the Supreme Court tells us in *Gladue*, because Indigenous traditions differ from Canadian criminal law in their view of how to respond to wrongdoing, often emphasising restorative justice and community-oriented processes of accountability. Consequently, punitive responses to crime may be culturally inappropriate and ineffective for some Indigenous offenders. *Gladue* (n 50) paras 70–4. See also *Ipeelee* (n 51) para 74.

54
*Gladue* (n 50) para 67.

55 ibid paras 69, 77.

56
*Ipeelee* (n 51) para 73.

57 The first two decades of the 21st century witnessed significant growth in the percentage of federal prisoners who are Indigenous. The reasons for this growth are more complicated than is typically acknowledged, however, because over that same period there has also been substantial growth in the Indigenous population, which has been partly a function of increased Indigenous self-identification. For further discussion, see Ewing and Kerr (n 48) 157 fn 4.

58 For a concise overview of some of these studies, see ibid 166–8.

59
*Ipeelee* (n 51) para 63.

60 ibid.

61 ibid paras 80–7.

62 Marie-Andrée Denis-Boileau and Marie-Ève Sylvestre, ‘*Ipeelee* and the Duty to Resist’ (2018) 51 UBC Law Review 548.

63 In both *Gladue* and *Ipeelee*, the Supreme Court summarily dismissed the idea that s 718.2(*e*) calls for ‘affirmative action’ or ‘reverse discrimination’. *Gladue* (n 50) paras 86–8; *Ipeelee* (n 51) para 71. Yet, in neither instance did the Supreme Court analyse different meanings or forms of affirmative action. Instead, it implied that it was simply rejecting politically charged language and the idea that s 718.2(*e*) requires an ‘automatic’ or merely ‘race-based’ discount at sentencing—rather than a searching, individualised inquiry into aspects of an Indigenous offender’s background circumstances relevant to a proportionate sentence. In any case, we are interested in a specific form of affirmative action, and in analytic clarity rather than the best interpretation of Canadian positive law or optimal rhetoric for judicial statecraft. One commentator has previously connected the *Gladue* principles to the idea of ‘affirmative action’ in criminal justice—understood, however, in terms of the application of restorative justice to the criminal law. May Lydia Yeh, ‘Restorative Justice, Affirmative Action Sentencing Legislation and the Canucks: Lessons from Our Northern Neighbor’ (2008) 7 Wash U Global Stud L Rev 661.

64 Criminal Code (n 49) s 718.1.

65
*Ipeelee* (n 51) para 77 (quoting *Gladue* (n 50) para 69).

66 In previous work, a coauthor and I rejected this ‘deflationary view’ of s 718.2(*e*) and *Gladue* as inadequate doctrinally as well as normatively. See Ewing and Kerr (n 48) 170–2.

67 See *Ipeelee* (n 51) para 65.

68 For a prior discussion of this dilemma surrounding the interpretation of s 718.2(*e*) and *Gladue*, and an attempt to conceptualise their distinctive remedial content partly in terms of their implicit instruction to judges to think more capaciously about the fairness of Indigenous offenders’ opportunities to avoid their crimes, see Ewing and Kerr (n 48) 170–9.

69 As a doctrinal matter, there is implicit support for such a view in *Ipeelee*. In that case, the Supreme Court clarified that life circumstances connected to an offender’s Indigenous heritage can still affect what sentence would be appropriate even if the offender is not able to trace a clear causal connection between those life circumstances and the offender’s unlawful conduct. *Ipeelee* (n 51) paras 81–3.

70
*Gladue* (n 50) para 68 (emphasis added).

71 In a move that has sometimes been referred to as extending a version of *Gladue* to bail, in 2019 Parliament added to the Criminal Code a provision declaring that the principle of restraint in the use of pretrial detention should be applied with ‘particular attention to the circumstances’ not only of Indigenous accused, but also of any ‘accused who belong to a vulnerable population that is overrepresented in the criminal justice system and that is disadvantaged in obtaining [pretrial] release’. Criminal Code (n 49) s 493.2.

